# Negative bias in encoding and recall memory in depressed patients with inadequate response to antidepressant medication

**DOI:** 10.1007/s00213-025-06857-0

**Published:** 2025-07-11

**Authors:** Fitri Fareez Ramli, Nisha Singh, Luca M. Villa, Shona Waters, Catherine J. Harmer, Philip J. Cowen, Beata R. Godlewska

**Affiliations:** 1 Clinical Psychopharmacology Research Group, Department of Psychiatry, University of Oxford, Warneford Hospital, Oxford, OX3 7JX UK; 2 Oxford Health NHS Foundation Trust, Oxford, UK; 3 Department of Pharmacology, Faculty of Medicine, Universiti Kebangsaan Malaysia, Kuala Lumpur, 56000 Malaysia; 4 Department of Paediatrics, University of Oxford, Oxford, OX3 9DU UK; 5 QYNAPSE SAS, 2-10 Rue d’Oradour-sur-Glane, Paris, 75015 France

**Keywords:** Antidepressants, Depression, Emotional biases, Emotional processing, Recall memory

## Abstract

**Rationale:**

Cognitive theories propose that negative biases in emotional processing contribute to the maintenance of depressive states. Previous studies reported that acute antidepressant treatment in depressed patients reversed negative emotional biases. However, studies addressing the differences in emotional processing between healthy volunteers and clinically depressed patients with inadequate response to standard antidepressant treatments are limited.

**Objectives:**

To investigate the differences in emotional processing domains between depressed patients with inadequate response to current antidepressant treatment and healthy controls.

**Methods:**

Fifty-four medicated patients with major depression and 45 age- and sex-equated healthy volunteers were tested using the Oxford Emotional Testing Battery.

**Results:**

There was no difference between the two groups in the accuracy of recognising emotional facial expressions. However, there was a significant difference in the pattern of response times in an emotional categorisation task (F_1,97_ = 6.44, *p* = 0.013, partial η^2^ = 0.017) where healthy controls had faster responses towards positive than negative self-referent words (95%CI: -0.291 – -0.054, *p* = 0.005). In contrast, patients had no significant differences in reaction time for categorizing positive and negative self-referent descriptors. There was also a significant group interaction in an emotional memory task (F_1,91_ = 7.90, *p* = 0.006, partial η^2^ = 0.080) where healthy volunteers recalled significantly more positively valenced words than depressed patients (95%CI: -2.104 – -0.168, *p* = 0.022).

**Conclusions:**

Depressed patients with inadequate responses toward antidepressants had negative biases in emotional categorisation and emotional memory. These psychological abnormalities may represent targets for treatment in patients with difficult-to-treat depression.

**Supplementary Information:**

The online version contains supplementary material available at 10.1007/s00213-025-06857-0.

## Introduction

Emotional processing is one of the cognitive domains affected by depression. Many cognitive theories propose negative biases in emotional processing as a contributing factor to the initiation and maintenance of depressive states (Beck 1979; Disner et al. 2011). Previous studies reported that unmedicated depressed patients had lower accuracy in recognizing positive versus negative facial expressions, longer latencies to respond when categorizing positive self-referent words, and lower recall rates of positive self-referent words (Disner et al. 2011). Interestingly, acute antidepressant treatment reversed these emotional biases; this effect was seen long before the onset of clear clinical benefit (Harmer et al. 2009). A previous study evaluating selective attention towards visual images of positive, dysphoric, threatening, or neutral valences reported higher selective attention towards positive visual stimuli in healthy volunteers and medicated depressed patients than in unmedicated depressed patients. Further, the differences between medicated depressed patients and healthy volunteers were not significant. Interestingly, both depressed groups had no significant differences in Beck Depression Inventory-II scores. It could be speculated that medication use was associated with positive bias in medicated depressed patients. However, a small sample size (21 medicated patients) might hinder the detection of small differences between medicated depressed patients and healthy volunteers, as medications might have shifted some degree of emotional processing in facial emotion recognition (Wells et al. 2014).

A meta-analysis of 22 studies assessing facial emotional recognition in depressed patients reported significant deficits in recognizing emotions of fear, happiness, surprise, disgust and anger with preservation of recognition toward sadness. A subgroup analysis reported that medicated and unmedicated depressed patients did not differ significantly in recognizing happy or sad facial expressions (Dalili et al. 2015). The limitation of this study was the lack of subgroup analysis comparing non-responders and healthy volunteers; it was therefore unclear whether non-responders to treatment and healthy volunteers differed in terms of facial expression recognition. Also, the meta-analysis focussed only on the accuracy of recognizing facial emotion. Other aspects, such as misclassification and response biases were not evaluated. Further, the meta-analysis was limited to one emotional processing domain (facial emotion recognition), and other emotional processing aspects, such as emotional memory, were not evaluated.

It is, therefore, uncertain whether the emotional processing of patients who are clinically unresponsive to antidepressant treatment differs significantly from that of non-depressed volunteers. The aim of this study was to explore this issue. The evaluations of various emotional processing domains (facial expression recognition, emotional categorization, emotional recall memory) can give more insight into the state of emotional processing in depressed patients unresponsive to medication and could provide novel targets for treatment.

## Method

We conducted a case-control study to investigate the differences in emotional processing domains between depressed patients with inadequate response to current antidepressant treatment and age- and sex-equated healthy controls. The study was approved by the National Research Ethics Service Committee (NRES), South-Central Oxford (ID:20/SC/0151). Both patients and healthy volunteers were recruited from the general population (through various platforms, including websites, newspapers and social media) and patients additionally through primary and secondary (mental health) care. Informed written consent was obtained from all participants before the study. Using the accuracy of detecting happy facial expressions (the Facial Expression Recognition Task (FERT) from the Emotional Testing Battery) from a previous study (Harmer et al. 2009), the calculated required sample size was 24 (12 participants in each arm) to detect a significant difference between the two groups. We included 45 healthy volunteers to equate the patient group who later underwent a drug study with ebselen (see below).

Potential patients with major depressive disorder (MDD) and healthy volunteers were screened using the Structured Clinical Interview of Diagnostic and Statistical Manual (DSM) −5 (SCID-5) and the Hamilton Depression Rating Scale-17 (HAM-D). The diagnosis of psychiatric disorders (MDD and other psychiatric disorders) was determined by a consultant psychiatrist (BRG) using the SCID-5. We included MDD patients who were still depressed with HAM-D scores of at least 14, despite being on adequate doses for at least four weeks. Patients who failed at least one treatment with antidepressant were included. The current study was part of the study investigating the effect of ebselen (a lithium-mimetic agent) on emotional processing as an augmenting agent (further reading, refer to (Ramli et al. 2024). We did not restrict inclusion to those who failed to respond to specific antidepressants as we wanted to include a patient group whose next treatment step is often augmentation with another medication, such as lithium. The exclusion criteria for patients with MDD were any current or history of schizophrenia, bipolar mood disorder and emotionally unstable personality disorder; being clinically suicidal; current or history of electroconvulsive therapy; current failure to respond to standard pharmacological augmentation therapy; history of substance dependence over the past six months; and participation in a study involving emotional processing tasks or interventional medication within the past three months. Healthy controls were required to be free from any current or history of major psychiatric disorder. Also, healthy controls had not participated in a study involving emotional processing tasks or interventional medication within the past three months.

Our primary outcomes were emotional processing tasks measured using the Oxford Emotional Testing Battery (ETB). This battery consisted of five tasks; including the FERT, Emotional Categorisation Task (ECAT), Facial Dot-Probe Task (FDOT), Emotional Recall Task (EREC) and Emotional Recognition Memory Task (EMEM) (Harmer et al. 2004; Murphy et al. 2008).

Generally, the FERT consists of 250 facial expressions of six basic emotions (sad, fear, happy, surprise, disgust, anger) with varying intensity ranging from 10–100%. There were 40 faces in each emotion and 10 neutral faces (0% intensity). One face was presented at a time on the laptop screen, and participants were asked to respond as accurately and quickly as possible by pressing one of the labelled buttons (sad, fear, happy, surprise, disgust, anger, neutral) on the keyboard. The ECAT consisted of positive or negative self-referent words presented one at a time and participants were required to choose whether they would like or dislike to be described using those words. The FDOT assessed the attentional vigilance score, whereby two faces (one neutral and one emotional- either happy or fear) were presented after a central ‘X’ mark disappeared. Two dots were then presented either at the top or the bottom of the face following the disappearance of the faces, and participants were asked to indicate the orientation of the dots (vertical or horizontal). The participants were then asked to recall the words from the ECAT within four minutes. EMEM was the last task, where participants were presented with self-referent words and were asked to indicate whether the word was presented previously in the ECAT or not. We measured accuracy (FERT, ECAT, EREC, EMEM), reaction time (FERT, ECAT, EMEM), misclassification (FERT, EREC, EMEM), d prime (FERT, EMEM) and beta (FERT, EMEM) for ETB tasks. D prime and beta are measures of response bias. We calculated d prime and beta values using the following formula: d prime = = 0.5 + ((y – x)(1 + y – x)/4y (1 – x)), 0 < d’ < 1; beta = y(1 – y) – x(1 – x)/y(1 – y) + x(1 – x), −1 < ß < 1 (x is a false alarm (proportion of incorrect responses), and y is a hit rate (proportion of correct responses). We assessed clinical measures of depression using the Montgomery Asberg Depression Rating Scale (MADRS) and 16-item Self-Report Quick Inventory of Depressive Symptomatology (QIDS-SR-16) and anxiety with the 7-item Generalized Anxiety Disorder (GAD-7) in both participant groups.

All analyses were conducted using IBM SPSS statistics, version 29. Continuous variables were reported in means and standard error of means (SEMs), and categorical data were reported in counts and percentages. Comparison between groups was conducted using independent and paired *t*-tests, while categorical variables were analysed using a chi-squared test. A two-way analysis of variance (ANOVA) with emotion as a within-subject factor and group (patients vs. healthy volunteers) as a between-subject factor was used to analyse parameters from the FERT, ECAT, EREC, and EMEM tasks. We utilised three-way and two-way ANOVAs to analyse the FDOT, whereby emotions and conditions (masked or unmasked) were within-subject factors and group was a between-subject factor. A significant interaction was followed up with a post-hoc independent *t*-test. For reaction time, a post-hoc paired *t*-test was conducted if there was a significant group interaction. Sub-analyses comparing two groups (SSRI-only vs. healthy volunteers and 1–2 vs. > 2 failed antidepressants) were also conducted using ANOVAs and post-hoc *t*-tests if there were any significant interactions. A Pearson’s product-moment correlation was used to test for correlations between clinical measures (HAM-D, MADRS, QIDS-SR-16, GAD-7) and ETB task if there was a significant interaction in the two-way ANOVA. The correlation analyses between clinical measures and ETB tasks were conducted separately for patients and healthy volunteers. A p-value of < 0.05 was considered statistically significant.

## Results

We included 54 clinically depressed patients, not responsive to their current antidepressant treatments and 45 age- and sex-equated healthy volunteers. No significant differences in age (*t*(97) = −1.097, 95%CI: −8.853–2.549, Cohen’s *d* = −0.221, *p* = 0.275) and sex (*p* = 0.635 (*χ* ^2^)) were observed between groups. As expected, the scores of HAM-D (*t*(59.6) = 30.96, 95%CI: 17.540–19.964, Cohen’s *d* = 5.753, *p* < 0.001), MADRS (*t*(60) = 25.15, 95%CI: 23.714 − 27.812, Cohen’s *d* = −4.675, *p* < 0.001), QIDS-SR-16 (*t*(84.4) = 18.24, 95%CI: 13.420–16.706, Cohen’s *d* = 3.501, *p* < 0.001), and GAD-7 (*t*(61) = 12.81, 95%CI: 7.767–10.640, Cohen’s *d* = −2.385, *p* < 0.001) were significantly higher in patients than healthy volunteers (Table 1). SSRIs (70.1%) were the most common drug class used by MDD patients in the current study, followed by atypical antidepressants (16.4%) and SNRIs (9.0%) (Table 2).

**Table 1:** Characteristics and clinical measures of participants included in the study (Means ± SEMs)

Variables	Patients (*n* = 54)	Healthy volunteers (*n* = 45)
Age (years)	38.5 *±* 14.2	41.7 *±* 14.3
Sex	Female: 36 (66.7%); Male: 18 (33.3%)	Female: 32 (71.1%); Male: 18 (28.9%)
HAM-D	19.5 *±* 4.3	0.7 *±* 1.0
MADRS	27.1 *±* 7.3	1.3 *±* 1.7
QIDS-SR-16	18.4 *±* 5.2	3.3 *±* 2.8
GAD-7	10.1 *±* 5.1	0.9 *±* 1.3
Duration of illness	15.1 *±* 1.7 years < 2 years: 7.4% (range 0.8–1.5) *≥* 2 years: 92.6% (range 2–46 years)	
No of failed antidepressants	1–2: 34 (63.0%) > 2: 20 (37.0%)	
Current treatment		
Illness duration	8.9 *±* 1.4 years (0.3–40) < 2 years: 18.9% (range 0.3–1.5) *≥* 2 years: 81.1% (range 2–46 years)	
Antidepressants	SSRI only: 39 (72.2%) SSRI combined with other classes: 3 (5.6%) SNRI only: 2 (3.7%) SNRI combined with other classes: 2 (3.7%) Atypical: 6 (11.1%) TCA: 1 (1.9%) Serotonin modulators: 1 (1.9%)	-
Pharmacological treatment duration	44.2 *±* 6.9 months Range: 2–192 months	-
Talking therapy	Yes: 2 (3.7%) No: 52 (96.3%)	-

**Table 2:** Counts and percentages of overall antidepressants used by depressed patients (*n* = 54)

Antidepressants	Count	Percentage
Selective serotonin reuptake inhibitor		
Sertraline	23	34.3
Citalopram	13	19.4
Fluoxetine	7	10.4
Escitalopram	3	4.5
Paroxetine	1	1.5
Serotonin and norepinephrine reuptake inhibitor		
Venlafaxine	5	7.5
Duloxetine	1	1.5
Norepinephrine-dopamine reuptake inhibitor		
Bupropion	1	1.5
Atypical		
Mirtazapine	9	13.4
Agomelatine	2	3.0
Serotonin modulators		
vortioxetine	1	1.5
Tricyclic antidepressants		
Imipramine	1	1.5

### Depressed patients vs. healthy volunteers

**Fig. 1:**
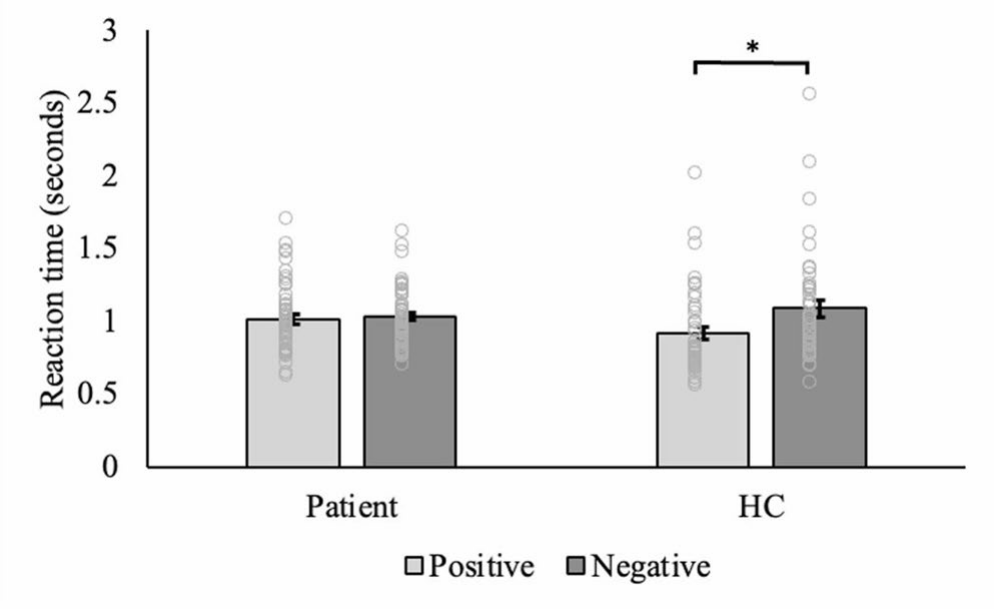
Means (± SEMs) reaction time in the ECAT task. There was a significant two-way ANOVA (group by valance) (F_1,97_ = 6.44, *p* = 0.013, partial η^2^ = 0.017). A follow-up paired *t*-test showed healthy controls were faster to respond towards positive (0.916 *±* 0.044) than negative self-referent words (1.089 *±* 0.056; *t*(44) = −2.936, *p* = 0.005)

A significant two-way ANOVA between group and valance was observed in the ECAT reaction time (F_1,97_ = 6.44, *p* = 0.013, partial η^2^ = 0.017). A follow-up paired *t*-test showed healthy controls were faster to respond towards positive (0.916 *±* 0.044) than negative self-referent words (1.089 *±* 0.056; *t*(44) = −2.936, 95%CI: −0.291 – −0.054, Cohen’s *d* = −0.438, *p* = 0.005) (Fig. 1). However, patients did not show any significant differences in reaction time for categorizing positive (1.013 + 0.031) and negative self-referent words (1.033 + 0.026; *t*(53) = −0.835, 95%CI: −0.070 – −0.029, Cohen’s *d* = −0.114, *p* = 0.407) (Fig. 1). We found no significant two-way ANOVA between group and valance in the ECAT accuracy (F_1,97_ = 1.68, *p* = 0.198, partial η^2^ = 0.017).

**Fig. 2:**
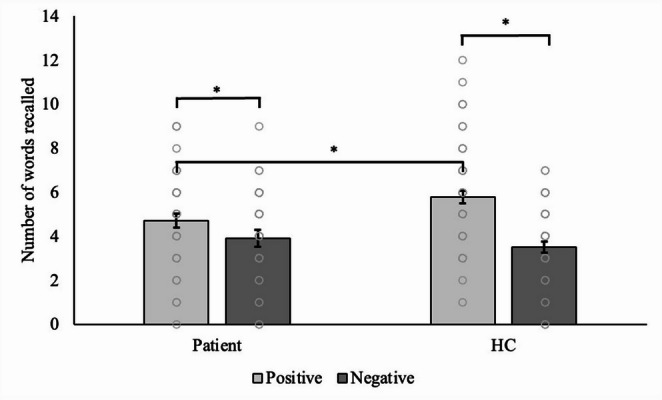
Means (± SEMs) number of positive and negative self-referent words recalled in the EREC task. There was a significant two-way ANOVA (group by valance) (F_1,91_ = 7.90, *p* = 0.006, partial η^2^ = 0.080). A follow-up independent *t*-test showed lower accuracy in recalling positive self-referent words in patients than in healthy volunteers (*p < 0.05)

We found a significant group-by-valance interaction in the EREC task accuracy (F_1,91_ = 7.90, *p* = 0.006, partial η^2^ = 0.080). A follow-up paired *t*-test (*t*(47) = 2.208, 95%CI: 0.070–1.513, Cohen’s *d* = 0.319, *p* = 0.032) showed that patients recalled more positive (4.71 *±* 0.314) than negative (3.92 *±* 0.277) self-referent words (Fig. 2). Similarly, healthy volunteers recalled more positive (5.84 *±* 0.376) than negative (3.62 *±* 0.281) self-referent words than healthy volunteers (*t*(44) = 6.159, 95%CI: 1.495–2.949, Cohen’s *d* = 0.918, p = < 0.001), but the effect size of healthy volunteers was higher than patients. When analysing based on emotion using a follow-up independent *t*-test (*t*(91) = −2.332, 95%CI: -−2.104 – −0.168, Cohen’s *d* = −0.484, *p* = 0.022), healthy volunteers (5.84 *±* 0.376) recalled more positive self-referent words than patients (4.71 *±* 0.314) (Fig. 2). Numerically, patients recalled more negative self-referent words (3.92 *±* 0.277) than healthy volunteers (3.62 *±* 0.281), but this was not statistically significant (*t*(91) = 0.745, 95%CI: −0.491–1.080, Cohen’s *d* = −0.155, *p* = 0.458). There was no significant group-by-valance interaction for the incorrect word recalls in the EREC task observed (F_1,91_ = 0.51, *p* = 0.479, partial η^2^ = 0.006).

No significant group interactions (between group and emotion or valance) in the FERT (accuracy, reaction time, misclassification, d prime, beta), ECAT (accuracy), FDOT (attentional vigilance scores), and EMEM (accuracy, reaction time, misclassification, d prime, beta) tasks were observed for any parameters (Supplementary Table S1).

### SSRI-only vs. healthy volunteers

We found a significant group-by-valance interaction (F_1,80_ = 4.09, *p* = 0.047, partial η^2^ = 0.049) in a subgroup analysis of the ECAT reaction time, with healthy controls showed faster respond towards positive (0.916 *±* 0.044) than negative self-referent words (1.089 *±* 0.056; *t*(44) = −2.936, 95%CI: −0.291 – −0.054, Cohen’s *d* = −0.438, *p* = 0.005). In contrast, no significant differences were observed in patients (*t*(36) = −1.089, 95%CI: −0.089–0.027, Cohen’s *d* = −0.179, *p* = 0.283).

Similarly, a subgroup analysis of the EREC task accuracy demonstrated a significant group-by-valance interaction (F_1,79_ = 8.95, *p* = 0.004, partial η^2^ = 0.102), with healthy controls showed higher recall for positive (5.84 *±* 0.376) than negative self-referent words (3.62 *±* 0.281; *t*(44) = 6.159, 95%CI: 1.495–2.949, Cohen’s *d* = 0.918, p = < 0.001). In contrast, no significant differences were observed in patients (*t*(35) = 1.532, 95%CI: −0.199–1.421, Cohen’s *d* = −0.255, *p* = 0.135).

We found no significant two-way interaction between group and emotion or valence in sub-analyses of SSRI-only compared to healthy volunteers for the same parameters (FERT (accuracy, reaction time, misclassification, d prime, beta), ECAT (accuracy), FDOT (attentional vigilance scores), and EMEM (accuracy, reaction time, misclassification, d prime, beta)) (Supplementary Table S2).

### 1–vs more than failed antidepressants

A sub-analysis between those who failed to 1–2 and > 2 antidepressants found no significant group-by-emotion or group-by-valance in all ETB parameters, including FERT (accuracy, reaction time, misclassification, d prime, beta), ECAT (accuracy, reaction time), EREC (accuracy and incorrect word recalls) and EMEM (accuracy, reaction time, misclassification, d prime, beta) (Supplementary Table S3). The only exception was the FDOT’s attentional vigilance score in which we found a significant group-by-valance interaction (F_1,52_ = 4.53, *p* = 0.038, partial η^2^ = 0.080). A post-hoc independent *t*-test demonstrated that patients who failed to 1–2 antidepressant treatments had higher attentional vigilance score towards fear in unmasked conditions (0.03 *±* 0.01) compared to those who failed more than two antidepressants (−0.02 *±* 0.02; *t*(52) = 2.113, 95%CI: 0.002–0.088, Cohen’s *d* = −0.596, *p* = 0.039).

### Correlation between clinical ratings and ETB parameters

**Fig. 3:**
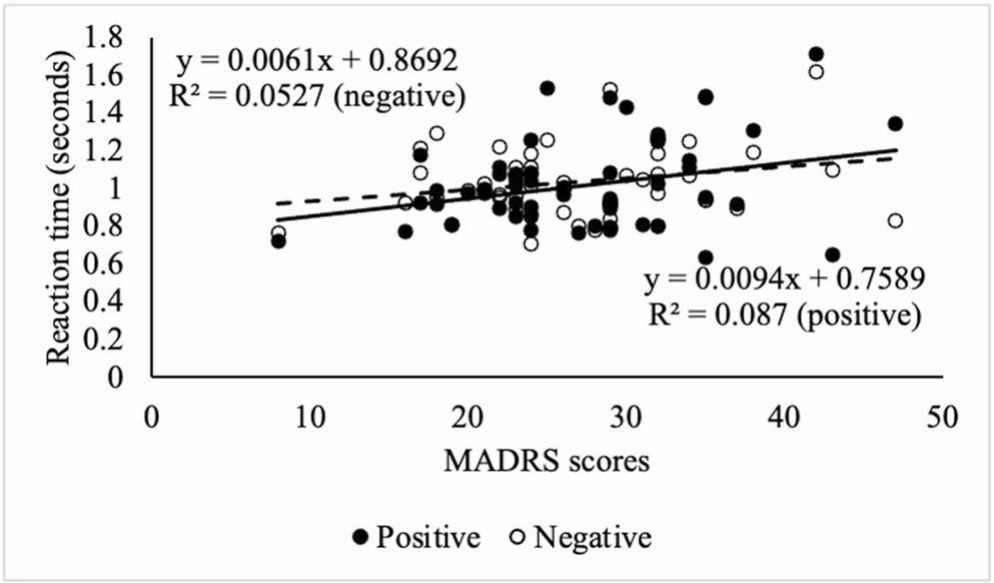
Correlation between MADRS scores and reaction time for categorizing positive (*r*(54) = 0.30, *p* = 0.03) and negative (*r*(54) = 0.23, *p* = 0.095) self-referent words in the patient group

There was a significant direct (the same direction) correlation between MADRS scores and reaction time of the ECAT (*r*(54) = 0.30, *p* = 0.03) for categorising positive self-reference words in the patient group (Fig. 3). There was a trend towards a direct correlation between MADRS scores and reaction time of the ECAT (*r*(54) = 0.23, *p* = 0.095) for categorising negative self-reference words in the patient group (Fig. 3, Supplementary Table S4). Also, we observed a trend towards a direct correlation between HAM-D scores and reaction time of the ECAT (*r*(54) = 0.23, *p* = 0.094) for categorising positive self-reference words in the patient group (Supplementary; Table S4, Figure S1).

There was no statistical significance for correlations between clinical measures of HAM-D, MADRS, QIDS-SR-16 and GAD-7 and the EREC accuracy (Supplementary; Table S2). However, there was a trend towards significance for an inverse (the opposite direction) correlation between GAD-7 scores and the accuracy of recalling positive self-referent words in the patient group (*r*(48) = −0.25, *p* = 0.09) (Supplementary; Table S2, Figure S2).

## Discussion

Compared to non-depressed controls, depressed patients taking antidepressant medication demonstrated negative biases in measures of emotional categorisation and emotional memory. We found a significant group-by-valance interaction in the reaction time of categorizing self-referent words. Healthy controls had faster responses towards positive than negative self-referent words, but patients had no significant differences in reaction time for categorizing positive and negative self-referent descriptors. Also, there was a significant group-by-valance interaction in recalling self-referent words, with patients recalling fewer positive words than healthy volunteers. The number of negative self-referent words recalled was also numerically higher in patients than in healthy volunteers, but the difference did not reach statistical significance. Similar findings were observed in the subgroup analysis of those who were taking SSRI-only currently compared to healthy volunteers. Interestingly, longer reaction time for categorising positive self-referent words were observed with increasing depression severity, as measured by MADRS and HAM-D (a trend) in the patient group. For the categorization of negative self-referent words, the reaction time in the patient group was longer with increasing severity, but the result did not reach statistical significance. This might indicate a general psychomotor slowing in depressed patients. We found no significant differences in the FERT, FDOT and EMEM tasks. Overall, our findings suggest that some negative biases in specific aspects of emotional processing were associated with depressive states in patients with inadequate responses to antidepressant therapy. We hypothesized that these negative biases might contribute to the maintenance of the disorder (Pringle and Harmer 2015).

Previous studies reported that the antidepressant drug, reboxetine (given in a single dose of 4 mg), increased recognition of happy facial expressions in healthy volunteers and unmedicated depressed patients (Harmer et al. 2009). Interestingly, a 7-day treatment with reboxetine or citalopram reduced the accuracy of recognizing negative facial expressions of anger (reboxetine), fear (both), and disgust (citalopram) in healthy volunteers (Harmer et al. 2004). Regarding emotional categorization, acute treatment with reboxetine significantly reduced the reaction time for categorizing positive self-referent words in healthy volunteers (single and repeated doses) and unmedicated depressed patients (single dose) (Harmer et al. 2004, 2009). The recall of positive self-referent words increased after acute treatment with reboxetine (single dose and repeated dose) and citalopram (repeated doses) in healthy volunteers (both drugs) and unmedicated depressed patients (reboxetine) (Harmer et al. 2004, 2009). These findings indicate that acute antidepressant treatments produce positive biases in aspects of emotional processing very early in treatment, prior to the expected onset of clinical improvement.

Interestingly, our study did not find any significant differences between medicated depressed patients and controls in emotional facial recognition tasks, which corroborated the study of Wells et al. (2014). The exact reason for this is unknown. We speculated that current antidepressant treatments might normalise the processing of emotional visual stimuli, even when clinical therapeutic responses were not. Previous studies proposed the potential role of the emotional testing battery in predicting later clinical response of antidepressants. Two studies reported that the early changes in the FERT (1–2 weeks) predicted clinical responses at 4–6 weeks (Browning et al. 2019; Tranter et al. 2009). Perhaps, an early positive shift in facial expression recognition might be essential (but not sufficient) for a successful response to antidepressant therapy. Early clinical responses (> 50%) within the 1–2 weeks of treatment might be needed to achieve subsequent treatment remission, as reported by Fernandes and colleagues for sertraline, venlafaxine, and mirtazapine (Fernandes et al. 2024). Baseline clinical and biological characteristics may also be crucial for response, or the lack thereof, to antidepressant treatments. For example, lower severity levels of depression, older age, the lower baseline activity of pregenual anterior cingulate cortex to sad versus happy faces, as well as a direct correlation in functional connectivity between dorsal anterior cingulate cortex and dorsolateral prefrontal cortex, were shown as predictors for poorer treatment response (Cukor et al. 2024; Godlewska et al. 2018; Hieronymus et al. 2019; Zhukovsky et al. 2025). While this is an interesting and clinically relevant issue, we were unable to assess such differences in our investigation because we included only people who were not responsive to antidepressant treatments.

Our subgroup analysis comparing patients who failed 1–2 and > 2 antidepressants reported no significant differences in FERT parameters, which may suggest that the number of failed treatments is not a determining factor for differences in this emotional processing domain. Another possibility is that our sample size was insufficient to detect the differences between groups in facial expression recognition. Dalili and co-workers proposed that 1230 participants (615 cases and 615 controls) were required to detect the association based on their power analysis in their meta-analysis of facial emotion recognition.

Our findings indicate that clinically depressed patients taking antidepressant medication had negative biases in self-referential emotional judgement and memory. The outcomes suggest that emotional memory might be useful as a sensitive measure of emotional processing bias in the presence of clinical depression and ongoing antidepressant treatment. Negative memory biases in depressed patients might be attributed to neurobiological changes, including suppressed hippocampal neurogenesis, inhibition of midbrain dopaminergic neurons and over-sensitization of the amygdala (Dillon and Pizzagalli 2018). Although antidepressants have been reported to reverse structural changes in the hippocampus (Boldrini et al. 2013), the evidence regarding the differential effects of responder status is still scarce.

The study has several limitations. We did not have measures of emotional processing in the depressed patients prior to their antidepressant treatment. Therefore, we cannot exclude the possibility that these particular depressed patients may have lacked negative biases in facial expression recognition even before they received antidepressants. The case-control design of the current study did not allow us to make a causal inference. It would take a larger number of depressed patients using a prospective design to explore the effects of ineffective antidepressants on emotional processing domains. The sample size was relatively small and the study may, therefore, have lacked the power to detect particularly subtle differences between patients and healthy controls in some aspects of emotional processing. A larger sample size in a future study will allow for subgroup analysis of the effects of individual or specific classes of antidepressants on emotional processing. For instance, selective serotonin reuptake inhibitors and serotonin-norepinephrine reuptake inhibitors were reported to have superior positive effects in a set of memory testing tasks than tricyclic antidepressants (Rosenblat et al. 2015). However, previous studies indicate that a range of conventional antidepressants produce positive shifts in emotional bias in both clinically depressed patients and healthy volunteers, (Arnone et al. 2009; Harmer et al. 2004, 2008, 2009; Tranter et al. 2009).

Depressed patients with inadequate responses towards antidepressants had negative biases in emotional categorisation and emotional memory compared to healthy volunteers. Developing psychological or pharmacological treatments to target these specific negative biases could represent potential treatment strategies for patients with difficult to treat depression. For instance, in preclinical studies, ketamine has been shown to exert its rapid antidepressant effect by reducing existing negative memory biases through altered neurotransmission in the medial prefrontal cortex (Stuart et al. 2015).

## Electronic supplementary material

Below is the link to the electronic supplementary material.

Supplementary Material 1 (DOCX 87.2 KB)

## Data Availability

All data are available on request.
