# Supplementary material for: Negative bias in encoding and recall memory in depressed patients with inadequate response to antidepressant medication
Source: Psychopharmacology (Berl). 2025 Jul 11;243(3):513–20. doi: 10.1007/s00213-025-06857-0 (PMC12979351; doi:10.1007/s00213-025-06857-0)
Supplement: Supplementary file 1 — Supplementary Material 1 (DOCX 87.2 KB) [file 213_2025_6857_MOESM1_ESM.docx]

Supplementary materials

Title: Persistent Negative Bias in Encoding and Recall Memory in Depressed Patients with Inadequate Response To Antidepressant Medication

Authors: Dr Fitri Fareez Ramli MD^1,2,3^, Dr Nisha Singh DPhil^4^, Dr Luca M Villa PhD^1,5^, Ms Shona Waters MSc^1^, Professor Catherine J Harmer DPhil^1,2^, Professor Philip J Cowen FRCPsych ^1,2^, Dr Beata R Godlewska PhD^1,2*^

Affiliations:

^1^ Psychopharmacology Research Group, Department of Psychiatry, University of Oxford, Warneford Hospital, OX3 7JX Oxford, UK

^2^Oxford Health NHS Foundation Trust.

^3^Department of Pharmacology, Faculty of Medicine, Universiti Kebangsaan Malaysia, Kuala Lumpur 56000, Malaysia.

^4^Department of Paediatrics, University of Oxford, Oxford OX3 9DU, UK.

^5^QYNAPSE SAS, 2-10 Rue d'Oradour-sur-Glane, 75015 Paris, France

Corresponding Author:

Dr Beata R Godlewska

Psychopharmacology Research Group, Department of Psychiatry, University of Oxford, Warneford Hospital, OX3 7JX Oxford, UK

Tel +44 (0)1865 618309
Fax +44 (0)1865 793101

Table S1. Analysis of variance for the Facial Expression Recognition Task (FERT), Emotional Categorisation Task (ECAT), Facial Dot-Probe Task (FDOT), and Emotional Recognition Memory Task (EMEM)

|  | F-statistics | p-value | partial η^2^ |
| --- | --- | --- | --- |
| FERT (Group x emotion) | | | |
| Accuracy | F_4.3,404.7_ = 1.30 | 0.266 | 0.014 |
| RT | F_1.7,155.9_ = 0.39 | 0.636 | 0.004 |
| Misclassification | F_2.6,239.7_ = 1.16 | 0.322 | 0.012 |
| D prime | F_4.1,378.1_ = 1.42 | 0.225 | 0.015 |
| Beta | F_2.2,207.1_ = 0.53 | 0.609 | 0.006 |
| ECAT (Group x emotion) | | | |
| Accuracy | F_1,97_ = 1.68 | 0.198 | 0.017 |
| FDOT | | | |
| Three-way | | | |
| Group x emotion x valence | F_1,97_ = 0.80 | 0.373 | 0.008 |
| Two-way | | | |
| Group x emotion | F_1,97_ = 0.64 | 0.428 | 0.007 |
| Group x condition | F_1,97_ = 0.05 | 0.822 | 0.001 |
| Emotion x condition | F_1,97_ = 2.82 | 0.097 | 0.028 |
| EMEM (Group x emotion) | | | |
| Accuracy | F_1,97_ = 1.46 | 0.230 | 0.015 |
| RT | F_1,97_ = 0.004 | 0.951 | 0.000 |
| Misclassification | F_1,97_ = 1.46 | 0.230 | 0.015 |
| D prime | F_1,97_ = 0.469 | 0.495 | 0.005 |
| Beta | F_1,97_ = 0.31 | 0.578 | 0.003 |

ECAT: Emotional Categorisation Task; EMEM: Emotional Recognition Memory Task; FERT: Facial Expression Recognition Task; FDOT: Facial Dot-Probe Task; RT: reaction time

Table S2. Correlations between clinical measures of HAM-D, MADRS, QIDS-SR-16, and GAD-7 and reaction times of the ECAT and accuracy of the EREC tasks.

|  | HAM-D | MADRS | QIDS-SR-16 | GAD-7 |
| --- | --- | --- | --- | --- |
| ECAT RT | | | | |
| Healthy controls | | | | |
| Positive | *r*(45) = -0.09, p = 0.55 | *r*(45) = -0.17, p = 0.27 | *r*(45) = 0.02, p = 0.90 | *r*(45) = -0.12, p = 0.42 |
| Negative | *r*(45) = 0.09, p = 0.56 | *r*(45) = -0.12, p = 0.44 | *r*(45) = 0.04, p = 0.80 | *r*(45) = 0.14, p = 0.35 |
| Patients | | | | |
| Positive | *r*(54) = 0.23, p = 0.09 | *r*(54) = 0.30, p = 0.03* | *r*(54) = 0.14, p = 0.30 | *r*(54) = 0.17, p = 0.22 |
| Negative | *r*(54) = 0.06, p = 0.64 | *r*(54) = 0.23, p = 0.095 | *r*(54) = 0.13, p = 0.34 | *r*(54) = -0.003, p = 0.99 |
| EREC Accuracy | | | | |
| Healthy controls | | | | |
| Positive | *r*(45) = 0.05, p = 0.77 | *r*(45) = 0.01, p = 0.97 | *r*(45) = -0.09, p = 0.56 | *r*(45) = -0.11, p = 0.47 |
| Negative | *r*(45) = -0.06, p = 0.70 | *r*(45) = 0.02, p = 0.88 | *r*(45) = -0.08, p = 0.62 | *r*(45) = -0.04, p = 0.81 |
| Patients | | | | |
| Positive | *r*(48) = -0.09, p = 0.54 | *r*(48) = -0.07, p = 0.62 | *r*(48) = 0.01, p = 0.93 | *r*(48) = -0.25, p = 0.09 |
| Negative | *r*(48) = 0.02, p = 0.90 | *r*(48) = -0.12, p = 0.40 | *r*(48) = 0.07, p = 0.63 | *r*(48) = -0.01, p = 0.95 |

ECAT: Emotional Categorisation Task; EREC: Emotional Recall Task; GAD-7: 7-item Generalized Anxiety Disorder; HAM-D: Hamilton Depression Rating Scale; MADRS: Montgomery Asberg Depression Rating Scale; QIDS-SR-16: 16-item Self-Report Quick Inventory of Depressive Symptomatology. *p-value < 0.05


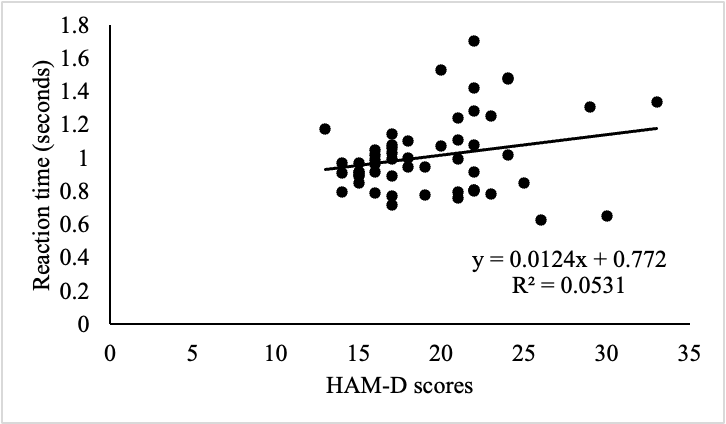


Figure S1. Correlation between HAM-D scores and reaction time for categorizing positive self-referent words (*r*(54) = 0.23, p = 0.09) in the patient group.


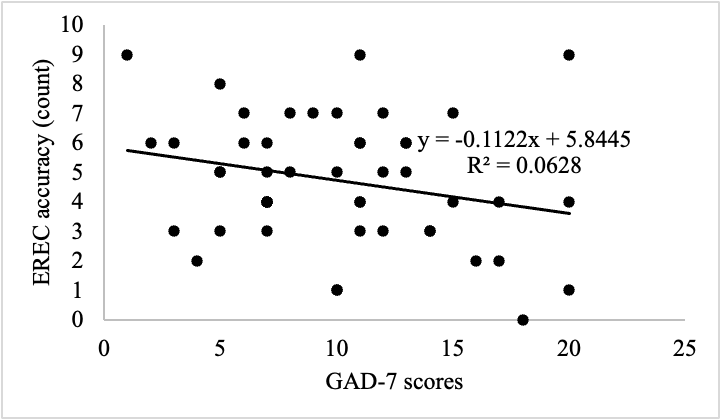


Figure S2. Correlation between GAD-7 scores and EREC accuracy (counts) for categorizing positive self-referent words (*r*(48) = -0.25, p = 0.09) in the patient group.
